# Cerebral and ocular toxoplasmosis related with IFN-γ, TNF-α, and IL-10 levels

**DOI:** 10.3389/fmicb.2014.00492

**Published:** 2014-10-13

**Authors:** Cristina S. Meira, Vera L. Pereira-Chioccola, José E. Vidal, Cinara C. Brandão de Mattos, Gabriela Motoie, Thais A. Costa-Silva, Ricardo Gava, Fábio B. Frederico, Luiz C. de Mattos, Toxoplasma Groups

**Affiliations:** ^1^Centro de Parasitologia e Micologia do Instituto Adolfo LutzSão Paulo, SP, Brazil; ^2^Departamento de Neurologia, Instituto de Infectologia Emílio RibasSão Paulo, SP, Brazil; ^3^Laboratório de Imunogenética, Departamento de Biologia Molecular, Faculdade de Medicina de São José do Rio PretoSão José do Rio Preto, SP, Brazil; ^4^Ambulatório de Oftalmologia, Fundação Faculdade Regional de Medicina, Hospital de BaseSão José do Rio Preto, SP, Brazil

**Keywords:** *Toxoplasma gondii*, cerebral toxoplasmosis and AIDS, ocular toxoplasmosis, cytokines, immune response

## Abstract

This study analyzed the synthesis of Interferon gamma (IFN-γ), Tumor Necrosis Factor alpha (TNF-α), and Interleukin 10 (IL-10) in chronically infected patients which developed the symptomatic disease as cerebral or ocular toxoplasmosis. Blood from 61 individuals were divided into four groups: Cerebral toxoplasmosis/AIDS patients (CT/AIDS group) (*n* = 15), ocular toxoplasmosis patients (OT group) (*n* = 23), chronic toxoplasmosis individuals (CHR group) (*n* = 13) and healthy individuals (HI group) (*n* = 10). OT, CHR, and HI groups were human immunodeficiency virus (HIV) seronegative. The diagnosis was made by laboratorial (PCR and ELISA) and clinical subjects. For cytokine determination, peripheral blood mononuclear cells (PBMC) of each patient were isolated and stimulated *in vitro* with *T. gondii* antigen. IFN-γ, TNF-α, and IL-10 activities were determined by ELISA. Patients from CT/AIDS and OT groups had low levels of IFN-γ when were compared with those from CHR group. These data suggest the low resistance to develop ocular lesions by the low ability to produce IFN-γ against the parasite. The same patients, which developed ocular or cerebral toxoplasmosis had higher TNF-α levels than CHR individuals. High TNF-α synthesis contribute to the inflammatory response and damage of the choroid and retina in OT patients and in AIDS patients caused a high inflammatory response as the TNF-α synthesis is not affected since monocytes are the major source this cytokine in response to soluble *T. gondii* antigens. IL-10 levels were almost similar in CT/AIDS and OT patients but low when compared with CHR individuals. The deviation to Th2 immune response including the production of anti-inflammatory cytokines, such as IL-10 may promote the parasite's survival causing the tissue immune destruction. IL-10 production in *T. gondii*-infected brains may support the persistence of parasites as down-regulating the intracerebral immune response. All these indicate that OT and CT/AIDS patients produced low levels of IL-10 (Th2 response) and IFN-γ (Th1 response). They produced high TNF-α suggesting a high inflammatory response triggered by the parasite.

## Introduction

Lifelong infection with the obligate intracellular protozoan *Toxoplasma gondii* affects one-third of the human population globally (Weiss and Dubey, 2009). Although toxoplasmosis is asymptomatic in the majority of cases, *T. gondii* infection can have serious consequences in immunocompromised individuals and in cases of congenital infection. In the latter case, ocular infection, posterior retinochoroiditis, is the most frequent clinical manifestation of congenital and acquired toxoplasmosis (Delair et al., 2011; Olariu et al., 2011; Dubey et al., 2012).

The severity and prevalence of the disease vary greatly and is believed to be affected by the status of the host immune system (Garweg and Candolfi, 2009), the genotype of infective parasite strains (Pereira-Chioccola et al., 2009; Dubey et al., 2012), and the host genetic background (Mack et al., 1999; Sullivan and Jeffers, 2012).

While the physiopathological mechanisms that underlie retinal damage in ocular toxoplasmosis are yet not fully understood, the immune response might directly affect the pathogenesis of toxoplasmic retinochoroiditis and some cytokines have been shown to be fundamental to either control or block a protective response against *T. gondii* in experimental models (Talabani et al., 2010). Its importance is even greater in Brazil, where the prevalence and severity of ocular disease is higher than those reported in other regions of the world (Gilbert et al., 2008; Ferreira et al., 2014). In addition, reactivation of the latent infection occurs in approximately 30% of immunocompromised patients (Sarciron and Guerardi, 2000; Robert-Gangneux and Dardé, 2012).

Cerebral toxoplasmosis is the most common AIDS-related opportunistic infection of the central nervous system and the most common cause of focal deficits in human immunodeficiency virus (HIV)-positive patients (Pereira-Chioccola et al., 2009; Vidal and Oliveira, 2013). It has been suggested that the neurological involvement of HIV leads to an imbalance of the immune response and, as consequence, the reactivation of the latent infection occurs (Lin and Bowman, 1992). However, the understanding of the physiopathology of cerebral toxoplasmosis remains incomplete.

In immunocompetent individuals, the immune system controls multiplication of the parasites and stops their dissemination. In addition, it favors the transformation of tachyzoites into bradyzoites and eventually, the occurrence of tissue cysts. Cytokines have been shown to play an important role in the pathogenesis of toxoplasmosis (Costa-Silva et al., 2012a; Ghasemi et al., 2012; Sullivan and Jeffers, 2012).

Although occur alterations in levels of antibodies and cytokines during the reactivation of *T. gondii* infection (Beaman et al., 1992; Meira et al., 2008, 2011; Hoti and Tandon, 2011), the role and function of cytokines, in cellular mediation, in the humoral response, as well as their impaired action in AIDS patients remain today only partially understood. This study was aimed to analyze the synthesis of Interferon gamma (IFN-γ), Tumor Necrosis Factor alpha (TNF-α), and Interleukin 10 (IL-10) in chronically infected patients which developed the symptomatic disease as cerebral or ocular toxoplasmosis.

## Materials and methods

### Patients and samples

This case-control study was conducted for 17 months (June 2011 to November 2012). We analyzed blood samples from 61 individuals divided into four groups. Two groups were formed of chronically patients infected, who developed the symptomatic disease: The CT/AIDS (cerebral toxoplasmosis and AIDS) group was composed of clinical samples from 15 patients with cerebral toxoplasmosis and AIDS (53% male and 47% female) aged between 23 and 64 years old. The OT (ocular toxoplasmosis) group was composed of clinical samples from 23 patients with ocular toxoplasmosis (57% male and 43% female) aged between 15 and 76 years old. The other two groups, CHR (chronic toxoplasmosis individuals) and HI (healthy individuals) were established as controls. Clinical samples from 13 chronic individuals for toxoplasmosis (44% male and 56% female) aged between 25 and 59 years old composed the CHR group. Samples from 10 healthy individuals, seronegative for *T. gondii*, (*Toxoplasma* uninfected group) (47% male and 53% female) aged between 28 and 38 years old composed the HI group. The OT, CHR, and HI groups were seronegative for HIV.

The 15 HIV-infected patients were admitted and treated at the Emilio Ribas Institute of Infectious Diseases, a tertiary teaching hospital in São Paulo, Brazil. The clinical diagnosis of cerebral toxoplasmosis in HIV-infected patients was based on: (1) progressive neurological deficits; (2) contrast-enhancing mass lesion(s) on computed tomography and/or magnetic resonance imaging; and (3) successful clinical and radiological response to antiparasitic treatment within 2 weeks (Portegies et al., 2004; Vidal and Oliveira, 2013).

OT patients were admitted and treated at the Ophthalmology Outpatient Clinics from Fundação Faculdade Regional de Medicina—Hospital de Base, a tertiary teaching hospital in São José do Rio Preto, São Paulo, Brazil. They were clinically diagnosed as having toxoplasmic retinochoroiditis, and the clinical evaluation was performed under fundoscopic examination using indirect binocular ophthalmoscopy, 20D lens (Binocular Ophthalmoscope ID 10, Topcon Corporation, USA). The clinical diagnosis of ocular toxoplasmosis was based on retina visualization and the description of the characteristic, the site and the size of exudative lesions or scars (Mattos et al., 2011; Ferreira et al., 2014). In order to determine the minimally invasive laboratory diagnosis, as defined before (Colombo et al., 2005; Mattos et al., 2011), blood samples from Group CT/AIDS and OT patients were collected before or until the third day of the antiparasitic therapy for toxoplasmosis.

#### Ethical considerations

All patients provided written informed consent and the institutional review boards of Ethics Committees of the all Institutions approved this study.

#### T. gondii and antigen

*T. gondii* lysate antigen (TLA) was prepared as described before (Meira et al., 2008; Costa-Silva et al., 2012b). Tachyzoites (RH strain) from Vero cell cultures in serum-free medium were purified by filtration. Then, parasites were washed, suspended in phosphate-buffered saline (PBS) and lysed using glass beads by vortex for 8 cycles for 4 min with 2-min intervals. Parasite extract was centrifuged (3000 g) and dissolved in 0.3 M NaCl, and the protein concentration was determined at 280 nm by spectrometry in a NanoDrop ND100 (Thermo Scientific). TLA was used for determination of humoral and cellular response by ELISA. Tachyzoites were further used for DNA extraction.

### Serological diagnosis

ELISA were performed with microtiter polystyrene plates (flat bottom, low binding, Corning Incorporated, USA) as previously described (Meira et al., 2008; Mattos et al., 2011). Briefly, each well was incubated overnight at 4 C with TLA at a concentration of 1 μg/ml. The free binding sites were blocked by treating the wells with 5% skim milk-PBS and after 60 min, 50 μl each of serum sample (1:200 in 5% skim milk-PBS) were incubated for 60 min at 37°C. After washes with PBS-Tween 20, the wells were incubated for more 60 min at 37°C with a horseradish peroxidase-conjugated goat anti-human IgG (Sigma) and the substrate solution (0.1 M citric acid, 0.2 M Na_2_HPO_4_, 0.05% *o*-phenylenediamine, 0.1% H_2_O_2_) was added to each well. Color development was stopped by adding 50 μl of 4 N H_2_SO_4_. Absorbance values were measured in an ELISA Labsystens-Multiscan MS Plate Reader (Minneapolis, Minnesota, USA) with a 492-nm filter. The optical density (O.D.) cutoff was 0.148 at 492-nm wavelength as previously described (Meira et al., 2008). The absorbance values were subtracted from the background, and the arithmetic mean was calculated. O.D. results were transformed in ELISA-relative values (RV) that represent the ratio of the absorbance of each serum sample at an optical density of 492 nm to the cutoff value (serum O.D/cutoff O.D.). Values greater than 1.0 were considered reactive. Low RV was considered to be between 1 and 5; and high RV, above 6.

### Molecular diagnosis

The DNA of blood samples was extracted by QIAamp DNA Mini Kit (Qiagen), according to the manufacturer's instructions. DNA pellets were dissolved in ultra-pure water. As a positive control, DNA was extracted from tachyzoite pellets using the same kit. DNA purity was determined by the ratio of O. D. at 260 and 280 nm in a NanoDrop ND100 (Thermo Scientific). The amplifications were carried out with a kit purchased from Promega (Go Taq Green Master Mix). The PCR mix (12.5 μL) was composed of 1 unit of Taq DNA polymerase, 10 mM Tris-HCl, pH 8.5; 50 mM KCl; 1.5 mM MgCl_2_; and 200 mM of each dNTP. The reactions included The PCR mix, 5 μL of each DNA template and 25 pmol of each primer to a final volume of 25 μL. The primer pairs used were B22/B23, which amplified a 115-bp sequence from a specific repetitive region of B1 gene from *T. gondii* as target (Burg et al., 1989). To control the course of extraction and check for PCR inhibitors, all samples were assayed using β1/β2 (Mesquita et al., 2010), which amplified a 140 bp fragment of the human β-globulin gene. Each amplification run contained two negative controls (ultra-pure water and a negative DNA for toxoplasmosis) and one positive (DNA extracted from tachyzoite pellets). The thermal cycles and PCR products analyses were made exactly as described before (Colombo et al., 2005; Mesquita et al., 2010).

### Isolation of peripheral blood mononuclear cells (PBMC) and cytokine assays

About 10 ml of blood containing sodium heparin as anticoagulant collected from all patients and an equal volume of PBS (pH 7.2) was added to the blood and the peripheral blood mononuclear cells (PBMC) separated over Histopaque 1077 as per protocol described by Sigma-Aldrich. After isolation, cells were washed two times with PBS by centrifugation at 1000 *g* for 10 min and resuspended in RPMI-1640 medium (Sigma) containing L-glutamine, 2 g/L sodium bicarbonate, 10% heat-inactivated fetal calf serum and 50 mg/ml of streptomycin. The viability of the cells used in the experiments was always higher than 85%, as measured by trypan blue exclusion (Sigma). The cells were then plated at a density of 1 × 10^6^ cells per well into 48-well culture plates in a final volume of 500 μL.

PBMC were then stimulated with 1 μg/mL of TLA. Alternatively, as control for IFN-γ, TNF-α, and IL-10 experiments, cells were stimulated with 2 μg/mL of phytohemagglutinin (PHA). Culture medium was used in all experiments as negative control wells. PBMC cultures were maintained at 37°C in a 5% CO_2_ atmosphere and supernatants were collected after 24–48 h of stimulation. The supernatant was analyzed after 24 h for TNF-α and IL-10, and 48 h for IFN-γ activity.

The cytokine concentrations were determined using the Ready-Set-Go ELISA kit according to the manufacturer's instructions (Ebioscience, San Diego, California). The limit of sensitivity of the assays was 2 pg/mL for IL-10 and 4 pg/mL for IFN-γ and TNF-α.

### Data analysis

The concentration of each cytokine, per group of patients was determined by median (in pg/mL). The statistical analyses were made by the non-parametric *Mann–Whitney* test for comparison between different groups in the GraphPad Prism 5.0 software. *p*-value < 0.05 was considered to be statistically significant.

## Results

The first step was to establish the clinical and laboratory diagnosis of the patients from the both groups. As described in Table 1, the 15 patients from CT/AIDS group developed focal cerebral toxoplasmosis as they had CD4+ lymphocytes counts less than 100 cells/μl of blood. The values ranged from 4 to 84 cells/μl of blood. In addition, they had positive PCR for *T. gondii* in blood, high ELISA-RV in 67% of the patients and low, in 33% of them. Out of the 23 patients from OT group, the clinical and fundoscopic examination determined that 2 patients had active lesions, 2 had both, active lesions and scars; and 19, retinochoroiditis scars. PCR was positive in 48% the patients. Low ELISA-RV was shown in 96% (Table 1).

**Table 1:** **Patients with cerebral toxoplasmosis or ocular toxoplasmosis: clinical and laboratory diagnosis as well as cytokines levels after PBMC stimulation with *T. gondii* TLA antigen**.

	**CT Patients^a^(*n* = 15)**	**OT patients^a^(*n* = 23)**
Data of the sample collection (m/y)^b^	10/11–3/12	5/12–8/12
Median age (range)	43 (23–64)	63 (15–76)
Gender	8M – 7F	13M – 10F
Clinical diagnosis^c^	focal CT (100%)	Active RtC (14%)^d^ Active and RtC scar (2%) RtC scar (84%)
HIV serology	Pos (100%)	ND^e^
CD4^+^ lym counts^f^ (range)	4–84	ND
Positive PCR in blood (for *T. gondii* DNA)	100% (15/15)	48% (11/23)
Anti-*T. gondii* antibodies ELISA (RV)^g^	Pos (12.8–3.0)	Pos (5.7–1.0)
^h^IFN-γ—median (range)	71.47 (0–633.24)	155.30 (27.35–2630.29)
^h^TNF-α—median (range)	2564.01 (21.12–11253.62)	2352.00 (401.03–11453.71)
^h^IL10—median (range)	3011.50 (17.87–726.38)	580.60 (39.15–2656.17)

a *CT (cerebral toxoplasmosis), OT (ocular toxoplasmosis)*.

b *Date (month/year) of the collection*.

c *Diagnosis was defined by clinical, images and laboratory data as described in Materials and Methods Section*.

d *Active and retinochoroiditis (RtC) scars in the right and left eyes, respectively*.

e *Non-determined (ND)*.

f *Number of CD4+ T lymphocytes/μl of blood*.

g *ELISA are expressed in relative values (RV), which represent the ratio of the absorbance of each serum sample at an optical density of 492 nm to the cutoff value (serum O.D./cutoff O.D.). Values greater than 1.0 were considered reactive*.

h *IFN-γ, TNF-α, and IL10 are expressed in in pg/mL. Pos, positive*.

**Figure 1:**
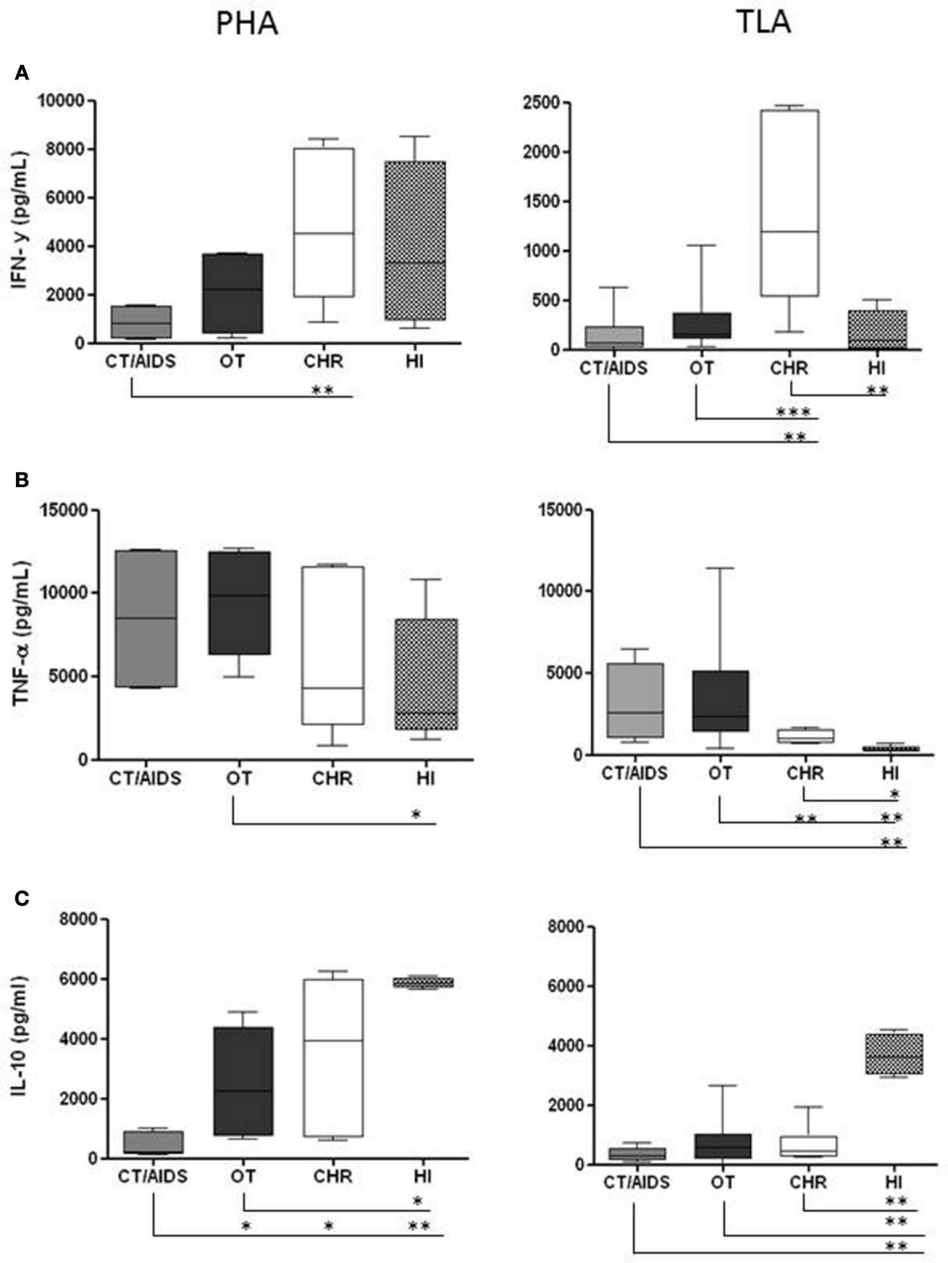
**Cytokine production from PBMC after PHA and TLA stimulation *in vitro***. PBMC (1 × 10^6^/well) from cerebral toxoplasmosis/AIDS patients (gray columns), ocular toxoplasmosis (black columns), chronic (white columns), and healthy individuals (dotted columns) stimulated with 2 and 1 μg/mL of PHA and TLA, respectively. Supernatants were collected after 48 h for IFN-γ **(A)** and 24 h for TNF-α **(B)** and IL-10 **(C)**. The cytokine levels were determined by ELISA. The horizontal line indicates the median, bars the 25 and 75% percentiles, and vertical lines the 10 and 90% percentiles. Comparison of reactivity between groups by *Mann–Whitney* test (95% confidence interval) at ^*^p < 0.05, ^**^p < 0.005, ^***^p < 0.0005.

The first part of the determinations of the cytokines was conducted in order to set up the conditions for PBMC cultures. Different TLA and PHA concentrations to stimulation of PBMC were tested (1, 2, 5, and 10 μg/mL) and the supernatants were tested after 24, 48 and 72 h of stimulation for each cytokine. After evaluation of these parameters, PBMC from CT/AIDS and OT patients; CHR and HI individuals were stimulated *in vitro* with 1 μg/mL of TLA as antigen. In parallel, as controls, the same cells were stimulated with 2 μg/mL of PHA and in the absence of the antigen. There was no proliferation in PBMC cultures in the absence of *T. gondii* antigen or PHA (data not shown). Next, IFN-γ, TNF-α, and IL-10 levels were evaluated by ELISA in the supernatant of PBMC collected from the four groups of patients. Median results of IFN-γ, TNF-α, and IL-10 levels after PBMC stimulation with TLA, per group of patient are shown in Table 1. Additionally, the median results of the each cytokine stimulated with PHA (as positive control) and TLA, per group, were analyzed and they were shown in details in Figure 1.

The IFN-γ data are shown in Figure 1A. PBMC from CT/AIDS and OT patients stimulated with PHA produced 840.60 pg/mL and 2205.00 pg/mL of IFN-γ, respectively. Differently, PBMC from CHR and HI individuals produced 4534.09 pg/mL and 3339.34 pg/mL, respectively. The differences between the results of CT/AIDS and CHR groups were statistically significant at *p* < 0.005.

PBMC from CHR individuals stimulated with TLA produced high IFN-γ amounts (1198.91 pg/mL). Nevertheless, PBMC from patients with CT/AIDS and OT produced low IFN-γ amounts (71.47 pg/mL and 155.30 pg/mL, respectively). PBMC from HI individuals produced 90.59 pg/mL of IFN-γ. The differences between results from CHR and CT/AIDS, CHR and HI individuals were statistically significant at *p* < 0.005, and between CHR and OT patients at *p* < 0.0005.

The TNF-α results are shown in Figure 1B. PBMC from individuals of all groups were able to produce large amounts of TNF-α using PHA as stimulus. CHR and HI individuals as well as CT/AIDS and OT patients, produced 4326.93 pg/mL, 2834.21 pg/mL, 8480.00 pg/mL and 9827.76 pg/mL of TNF-α, respectively. The differences between OT and HI were statistically significant at *p* < 0.05. Lower TNF-α level (992.71 pg/mL) produced from cells collected from CHR were shown when compared with CT/AIDS and OT patients (2564.54 pg/mL and 2352.52 pg/mL, respectively).

PBMC of HI individuals stimulated with TLA produced 329.60 pg/mL of TNF-α. The differences between the CHR and OT were statistically significant at *p* < 0.005 and between CT/AIDS and HI at *p* < 0.005, OT and HI at *p* < 0.005 and CHR and HI at *p* < 0.05.

The results of IL-10 levels are shown in Figure 1C. PBMC of CHR and HI individuals stimulated with PHA produced 3939.00 pg/mL and 5858.09 pg/mL while CT/AIDS and OT patients were able to produce 207.21 pg/mL and 2246.87 pg/mL, respectively. The differences between the CT/AIDS, OT and CHR were statistically significant at *p* < 0.05 and between CT/AIDS and HI at *p* < 0.005. In OT and HI groups the differences was at *p* < 0.05.

No differences in IL-10 levels were observed between PBMC collected from CHR individuals and CT/AIDS or OT patients (466.80 pg/mL, 311.50 pg/mL and 580.60 pg/mL, respectively). However, HI individuals produced high levels (3619.10 pg/mL). The differences between HI and CT/AIDS, HI and OT or HI and CHR were statistically significant at *p* < 0.0005.

## Discussion

This study evaluated the cellular response in PBMC samples from patients with cerebral toxoplasmosis and AIDS and patients with ocular toxoplasmosis using a TLA (Meira et al., 2013).

The clinical and laboratorial characteristics from the 38 chronically infected patients studied confirmed previous studies (Colombo et al., 2005; Mattos et al., 2011; Meira et al., 2013). The 15 patients from CT/AIDS group developed focal cerebral toxoplasmosis since they had low CD4+ lymphocytes counts. All patients had tachyzoites in blood since they had positive PCR. The *T. gondii* antigens produced high antibody production determined by ELISA-RV. The other group, which was composed of 23 patients with active lesions or retinochoroiditis scars, was a quite different. PCR results were positive in almost half of the patients but they had low ELISA-RV. No differences in IL-10 levels were observed between PBMC collected from CHR individuals.

The quality of the cells of all groups of individuals was in good conditions as showed in the PBMC stimulation with PHA. The importance of IFN-γ to resistance against *T. gondii* infection has been demonstrated, as this cytokine is responsible for regulation of *T. gondii* load and distribution in the eyes; essential mediator of the immune response to control *T. gondii* in the brain and to maintain the latency of chronic infection (Carruthers and Suzuki, 2007; Gaddi and Yap, 2007; Goldszmid et al., 2012; Nijhawan et al., 2013). However, patients develop cerebral toxoplasmosis after reactivation of latent infection during immunosuppression. In this study these patients (CT/AIDS group) had low levels of IFN-γ when were compared with those from CHR group (chronic infected individuals). The patients with ocular toxoplasmosis also had low levels of IFN-γ and confirm other studies that also shown IFN-γ levels were elevated in asymptomatic individuals compared with patients with acquired toxoplasmosis (Yamamoto et al., 2000; de-la-Torre et al., 2013). These data suggest that resistance or not to develop ocular lesions is associated with the ability to produce IFN-γ against the parasite. These data, yet, can be correlated with recent reported studies that severe ocular infections in South America due to highly variable *T. gondii s*trains and characterized by a completely different immune response pattern and much higher ocular parasite loads (de-la-Torre et al., 2013, 2014; Pfaff et al., 2014). The high levels of this cytokine found in CHR individuals can be explained by its release by parasite-specific T lymphocytes, which are required to prevent cyst reactivation during chronic infection (Sarciron and Guerardi, 2000).

On the other hand, the same patients, which developed ocular or cerebral toxoplasmosis had higher TNF-α levels than CHR individuals. This proinflammatory cytokine is directly involved in the regulation of tachyzoite growth. In addition, the balance between IFN-γ and TNF-α is the key mediators in triggering effector functions against *T. gondii* during both acute and chronic stages of infection (Denkers and Gazzinelli, 1998).

PBMC of OT patients also produced high TNF-α level after stimulus with TLA. These data are in agreement with other studies when suggested the susceptibility of OT patients with the production of TNF-α that contribute to the inflammatory response and damage of the choroid and retina (Wallace and Stanford, 2008; Talabani et al., 2010).

In AIDS patients, the TNF-α synthesis is not affected since monocytes are the major source this cytokine in response to soluble *T. gondii* antigens (Sarciron and Guerardi, 2000). These findings are in agreement with our data, as we found high levels TNF-α in CT/AIDS patients suggesting a high inflammatory response.

IL-10 levels in response to TLA were almost similar in CT/AIDS and OT patients but low when compared with CHR individuals. Similar results have been shown by others (Vallochi et al., 2002; de-la-Torre et al., 2014) and support the idea that IL-10 seems to be central to the induction of the permissive state seen in the eyes of South American OT patients (de-la-Torre et al., 2014). These data, also, could be explained by the deviation to Th2 immune response including the production of anti-inflammatory cytokines, such as IL-10, TGF-β, and IL-4 favor the parasite's survival. This strategy is required to maintain immune equilibrium in the eye preventing the tissue immune destruction (Neyer et al., 1997; Gaddi and Yap, 2007). IL-10 production in *T. gondii*-infected brains may support the persistence of parasites as down-regulating the intracerebral immune response. The ratio of IFN-γ/IL-10 response in the CHR and HI groups is related to the immunoregulatory effect as a mixed Th1/Th2 profile since these individuals have no impairment of lymphocytes and IFN-γ, which is a key cytokine for the control of infection (Neyer et al., 1997; Kumar et al., 1998). Similar data have been shown in chronic infected mice (Costa-Silva et al., 2012a).

All these data together indicate that OT and CT/AIDS patients produced low IL-10 (Th2 response) and IFN-γ (Th1 response). In addition, they produced high TNF-α suggesting a high inflammatory response triggered by the parasite. IFN-γ levels found in these patients may be due a global decrease of CD4+ T cells in CT/AIDS and a possible central tolerance to parasite antigens in those patients with ocular toxoplasmosis. These particular characteristics in Brazilian patients may be due to the nature of the infecting South American strains that have shown more severe those from other regions (Ajzenberg et al., 2004; Ferreira et al., 2011) or to the genetic susceptibility of the host or by both combined events.

The monitoring of the immune response to antigens involved in the different clinical forms of infection can provide valuable information that can help in understanding the mechanisms of immune system control over parasites.

## Author contributions

Vera Lucia Pereira-Chioccola and Cristina da Silva Meira designed the study and experiments; performed the data analysis, interpreted the data and wrote the manuscript. Cristina da Silva Meira, Thais Alves Costa-Silva, Ricardo Gava and Gabriela Motoie performed the laboratorial experiments (PCR, ELISA, Isolation of PBMC and cytokine assays). Cinara de Cássia Brandão de Mattos, Luiz Carlos de Mattos, José Ernesto Vidal and Gabriela Motoie revised critically the manuscript. Cinara de Cássia Brandão de Mattos and Luiz Carlos de Mattos interviewed the patients with ocular toxoplasmosis and collected the epidemiological data.

*FAMERP Toxoplasma Research Group* (Fábio Batista Frederico, Amanda Pires Barbosa, Plínio Pereira Martins Neto, Gildásio Castello Almeida Jr., and Mariana Previato) performed the inclusion of patients with ocular toxoplasmosis, sample collection, and develop the ophthalmological clinical evaluation and clinical analyses. *IIER Toxoplasma Research Group* (José Ernesto Vidal, Daniel Soares de Sousa Dantas, Tatiana Pimentel de Andrade Batista, Maria Jose Oliveira Kassab, Munir Bazzi, Daniel Paffili Prestes, Vanessa Levien Strelow, Adriana Weinfeld Massaia, Daniele Audi, Mariana Martins Lago, and Carlos Henrique Valente Moreira) performed the inclusion of patients with cerebral toxoplasmosis, sample collection, clinical diagnosis, acquisition data and follow-up of the patients.

All authors revised the manuscript, approved the final version submitted, published and agreement to be accountable for all aspects of the work in ensuring that questions related to the accuracy or integrity of any part of the work are appropriately investigated and resolved.

### Conflict of interest statement

The authors declare that the research was conducted in the absence of any commercial or financial relationships that could be construed as a potential conflict of interest.

## References

[B1] Ajzenberg D. Banuls A. L. Su C. Dumètre A. Demar M. Carme B. (2004). Genetic diversity, clonality and sexuality in Toxoplasma gondii.Int. J. Parasitol. 34, 1185–1196 10.1016/j.ijpara.2004.06.007 15380690

[B2] Beaman M. H. Wong S. Y. Remington J. S. (1992). Cytokines, *Toxoplasma* and intracellular parasitism. Immunol. Rev. 127, 97–117 10.1111/j.1600-065X.1992.tb01410.x 1506009

[B3] Burg J. L. Grover C. M. Pouletty P. Boothroyd J. C. (1989). Direct and sensitive detection of a pathogenic protozoan, *Toxoplasma gondii*, by polymerase chain reaction. J. Clin. Microbiol. 27, 1787–1792 2768467 10.1128/jcm.27.8.1787-1792.1989 PMC267672

[B4] Carruthers V. B. Suzuki Y. (2007). Effects of *Toxoplasma gondii* infection on the brain. Schizophr. Bull. 33, 745–751 10.1093/schbul/sbm008 17322557 PMC2526127

[B5] Colombo F. A. Vidal J. E. Penalva de Oliveira A. C. Hernandez A. V. Bonasser-Filho F. Nogueira R. S. (2005). Diagnosis of cerebral toxoplasmosis in AIDS patients in Brazil: importance of molecular and immunological methods using peripheral blood samples. J. Clin. Microbiol. 43, 5044–5047 10.1128/JCM.43.10.5044-5047.2005 16207959 PMC1248484

[B6] Costa-Silva T. A. Borges M. M. Galhardo C. S. Pereira-Chioccola V. L. (2012a). Immunization with excreted/secreted proteins in AS/n mice activating cellular and humoral response against *Toxoplasma gondii* infection. Acta Trop. 124, 203–209 10.1016/j.actatropica.2012.08.013 22940015

[B7] Costa-Silva T. A. Meira C. S. Frazzatti-Gallina N. Pereira-Chioccola V. L. (2012b). *Toxoplasma gondii* antigens: recovery analysis of tachyzoites cultivated in Vero cell maintained in serum free medium. Exp. Parasitol. 130, 463–469 10.1016/j.exppara.2012.01.005 22306070

[B8] Delair E. Latkany P. Noble A. G. Rabiah P. McLeod R. Brezin A. (2011). Clinical manifestations of ocular toxoplasmosis. Ocul. Immunol. Inflamm. 19, 91–102 10.3109/09273948.2011.564068 21428746

[B9] de-la-Torre A. Pfaff A. W. Grigg M. E. Villard O. Candolfi E. Gomez-Marin J. E. (2014). Ocular cytokinome is linked to clinical characteristics in ocular toxoplasmosis. Cytokine 68, 23–31 10.1016/j.cyto.2014.03.005 24787053 PMC4889015

[B10] de-la-Torre A. Sauer A. Pfaff A. W. Bourcier T. Brunet J. Speeg-Schatz C. (2013). Severe South American ocular toxoplasmosis is associated with decreased Ifn-γ/Il-17a and increased Il-6/Il-13 intraocular levels. PLoS Negl. Trop. Dis. 21:e2541 10.1371/journal.pntd.0002541 24278490 PMC3837637

[B11] Denkers E. Y. Gazzinelli R. T. (1998). Regulation and function of T-cell-mediated immunity during *Toxoplasma gondii* infection. Clin. Microbiol. Rev. 11, 569–588 9767056 10.1128/cmr.11.4.569 PMC88897

[B12] Dubey J. P. Lago E. G. Gennari S. M. Su C. Jones J. L. (2012). Toxoplasmosis in humans and animals in Brazil: high prevalence, high burden of disease, and epidemiology. Parasitology 139, 1375–1424 10.1017/S0031182012000765 22776427

[B13] Ferreira A. I. C. De Mattos C. C. Frederico F. B. Meira C. S. Almeida G. C. Jr. Nakashima F. (2014). Risk factors for ocular toxoplasmosis in Brazil. Epidemiol. Infect. 142, 142–148 10.1017/S0950268813000526 23507508 PMC3857107

[B14] Ferreira I. M. Vidal J. E. de Mattos C. C. de Mattos L. C. Qu D. Su C. (2011). *Toxoplasma gondii* isolates: multilocus RFLP-PCR genotyping from human patients in Sao Paulo State, Brazil identified distinct genotypes. Exp. Parasitol. 129, 190–195 10.1016/j.exppara.2011.06.002 21741380

[B15] Gaddi P. J. Yap G. S. (2007). Cytokine regulation of immunopathology in toxoplasmosis. Immunol. Cell Biol. 85, 155–159 10.1038/sj.icb.7100038 17228318

[B16] Garweg J. G. Candolfi E. (2009). Immunopathology in ocular toxoplasmosis: facts and clues. Mem. Inst. Oswaldo Cruz 104, 211–220 10.1590/S0074-02762009000200014 19430646

[B18] Ghasemi H. Ghazanfari T. Yaraee R. Owlia R. Hassan Z. M. Faghihzadeh S. (2012). Roles of IL-10 in ocular inflammations: a review. Ocul. Immunol. Inflamm. 20, 406–418 10.3109/09273948.2012.723109 23163602

[B19] Gilbert R. E. Freeman K. Lago E. G. Bahia-Oliveira L. M. Tan H. K. Wallon M. (2008). European Multicentre Study on Congenital Toxoplasmosis (EMSCOT). Ocular sequelae of congenital toxoplasmosis in Brazil compared with Europe. PLoS Negl. Trop. Dis. 2:e277 10.1371/journal.pntd.0000277 18698419 PMC2493041

[B20] Goldszmid R. S. Caspar P. Rivollier A. White S. Dzutsev A. Hieny S. (2012). NK cell-derived interferon-γ orchestrates cellular dynamics and the differentiation of monocytes into dendritic cells at the site of infection. Immunity 36, 1047–1059 10.1016/j.immuni.2012.03.026 22749354 PMC3412151

[B21] Hoti S. L. Tandon V. (2011). Ocular parasitoses and their immunology. Ocul. Immunol. Inflamm. 19, 385–396 10.3109/09273948.2011.626141 22106905

[B22] Kumar A. Angel J. B. Daftarian M. P. Parato K. Cameron W. D. Filion L. (1998). Differential production of IL-10 by T cells and monocytes of HIV-infected individuals: association of IL-10 production with CD28-mediated immune responsiveness. Clin. Exp. Immunol. 114, 78–86 10.1046/j.1365-2249.1998.00689.x 9764607 PMC1905077

[B23] Lin D. S. Bowman D. D. (1992). *Toxoplasma gondii*: an AIDS enhancing cofactor. Med. Hypotheses 39, 140–142 10.1016/0306-9877(92)90174-B 1334211

[B24] Mack D. G. Johnson J. J. Roberts F. Roberts C. W. Estes R. G. David C. (1999). HLA-class II genes modify outcome of *Toxoplasma gondii* infection. Int. J. Parasitol. 29, 1351–1358 10.1016/S0020-7519(99)00152-6 10579423

[B25] Mattos C. C. Meira C. S. Ferreira A. I. Frederico F. B. Hiramoto R. M. Jr G. C. (2011). Contribution of laboratory methods in diagnosing clinically suspected ocular toxoplasmosis in Brazilian patients. Diagn. Microbiol. Infect. Dis. 70, 362–366 10.1016/j.diagmicrobio.2011.02.002 21683267

[B26] Meira C. S. Costa-Silva T. A. Vidal J. E. Ferreira I. M. Hiramoto R. M. Pereira-Chioccola V. L. (2008). Use of the serum reactivity against *Toxoplasma gondii* excreted-secreted antigens in cerebral toxoplasmosis diagnosis in human immunodeficiency virus-infected patients. J. Med. Microbiol. 57, 845–850 10.1099/jmm.0.47687-0 18566142

[B27] Meira C. S. Vidal J. E. Costa-Silva T. A. Frazatti-Gallina N. Pereira-Chioccola V. L. (2011). Immunodiagnosis in cerebrospinal fluid of cerebral toxoplasmosis and HIV-infected patients using *Toxoplasma gondii* excreted/secreted antigens. Diagn. Microbiol. Infect. Dis. 71, 279–285 10.1016/j.diagmicrobio.2011.07.008 21907524

[B28] Meira C. S. Vidal J. E. Costa-Silva T. A. Motoie G. Gava R. Hiramoto R. M. (2013). IgG4 specific to *Toxoplasma gondii* excretory/secretory antigens in serum and/or cerebrospinal fluid support the cerebral toxoplasmosis diagnosis in HIV-infected patients. J. Immunol. Methods 395, 21–28 10.1016/j.jim.2013.06.005 23811152

[B29] Mesquita R. T. Ziegler A. P. Hiramoto R. M. Vidal J. E. Pereira-Chioccola V. L. (2010). Real-time quantitative PCR in cerebral toxoplasmosis diagnosis of Brazilian human immunodeficiency virus-infected patients. J. Med. Microbiol. 59, 641–647 10.1099/jmm.0.016261-0 20150319

[B30] Neyer L. E. Grunig G. Fort M. Remington J. S. Rennick D. Hunter C. A. (1997). Role of interleukin-10 in regulation of T cell dependent and T cell independent mechanisms of resistance to *Toxoplasma gondii*. Infect. Immun. 65, 1675–1682 9125546 10.1128/iai.65.5.1675-1682.1997 PMC175195

[B31] Nijhawan R. Bansal R. Gupta N. Beke N. Kulkarni P. Gupta A. (2013). Intraocular cysts of *Toxoplasma gondii* in patients with necrotizing retinitis following periocular/intraocular triamcinolone injection. Ocul. Immunol. Inflamm. 21, 396–399 10.3109/09273948.2013.810276 23876183

[B32] Olariu T. R. Remington J. S. McLeod R. Alam A. Montoya J. G. (2011). Severe congenital toxoplasmosis in the United States: clinical and serologic findings in untreated infants. Pediatr. Infect. Dis. J. 30, 1056–1061 10.1097/INF.0b013e3182343096 21956696

[B33] Pereira-Chioccola V. L. Vidal J. E. Su C. (2009). *Toxoplasma gondii* infection and cerebral toxoplasmosis in HIV-infected patients. Future Microbiol. 4, 1363–1379 10.2217/fmb.09.89 19995194

[B34] Pfaff A. de-la-Torre A. Rochet E. Brunet J. Sabou M. Sauer A. (2014). New clinical and experimental insights into Old World and neotropical ocular toxoplasmosis. Int. J. Parasitol. 44, 99–107 10.1016/j.ijpara.2013.09.007 24200675

[B35] Portegies P. Solod L. Cinque P. Chaudhuri A. Begovac J. Everall I. (2004). Guidelines for the diagnosis and management of neurological complications of HIV infection. Eur. J. Neurol. 11, 297–304 10.1111/j.1468-1331.2004.00856.x 15142222

[B36] Robert-Gangneux F. Dardé M. L. (2012). Epidemiology of and diagnostic strategies for toxoplasmosis. Clin. Microbiol. Rev. 25, 264–296 10.1128/CMR.05013-11 22491772 PMC3346298

[B37] Sarciron M. E. Guerardi A. (2000). Cytokines involved in Toxoplasmic encephalitis. Scand. J. Immunol. 52, 534–543 10.1046/j.1365-3083.2000.00817.x 11119257

[B38] Sullivan W. J. Jr. Jeffers V. (2012). Mechanisms of *Toxoplasma gondii* persistence and latency. FEMS Microbiol. Rev. 36, 717–733 10.1111/j.1574-6976.2011.00305.x 22091606 PMC3319474

[B39] Talabani H. Mergey T. Year H. Delair E. Brézin A. P. Langsley G. (2010). Factors of occurrence of ocular toxoplasmosis. A review. Parasite 17, 177–182 10.1051/parasite/2010173177 21073138

[B41] Vallochi A. L. Nakamura M. V. Schlesinger D. Martins M. C. Silveira C. Belfort R. (2002). Ocular toxoplasmosis: more than just what meets the eye. Scand. J. Immunol. 55, 324–328 10.1046/j.1365-3083.2002.01052.x 11967112

[B42] Vidal J. E. Oliveira A. C. (2013). AIDS-related cerebral toxoplasmosis in São Paulo State, Brazil: marked improvements in the highly active antiretroviral therapy-era but the challenges continue. Braz. J. Infect. Dis. 17, 379–380 10.1016/j.bjid.2012.10.030 23607923 PMC9427350

[B43] Wallace G. R. Stanford M. R. (2008). Immunity and *Toxoplasma* retinochoroiditis. Clin. Exp. Immunol. 153, 309–315 10.1111/j.1365-2249.2008.03692.x 18549442 PMC2527354

[B44] Weiss L. M. Dubey J. P. (2009). Toxoplasmosis: a history of clinical observations. Int. J. Parasitol. 39, 895–901 10.1016/j.ijpara.2009.02.004 19217908 PMC2704023

[B45] Yamamoto J. H. Vallochi A. L. Silveira C. Filho J. K. Nussenblatt R. B. Cunha-Neto E. (2000). Discrimination between patients with acquired toxoplasmosis and congenital toxoplasmosis on the basis of the immune response to parasite antigens. J. Infect. Dis. 181, 2018–2022 10.1086/315494 10837184

